# A conceptual framework for how structural changes in emerging acute substance use service models can reduce stigma of medications for opioid use disorder

**DOI:** 10.3389/fpsyt.2023.1184951

**Published:** 2023-09-27

**Authors:** Timothy D. Becker, Evan L. Eschliman, Ashish P. Thakrar, Lawrence H. Yang

**Affiliations:** ^1^Department of Psychiatry, New York-Presbyterian Hospital/Weill Cornell Medicine, New York, NY, United States; ^2^Department of Psychiatry, Columbia University Vagelos College of Physicians and Surgeons, New York, NY, United States; ^3^Department of Health, Behavior and Society, Johns Hopkins Bloomberg School of Public Health, Baltimore, MD, United States; ^4^Division of General Internal Medicine, Department of Medicine, Perelman School of Medicine at the University of Pennsylvania, Philadelphia, PA, United States; ^5^Department of Social and Behavioral Sciences, College of Global Public Health, New York University, New York, NY, United States; ^6^Department of Epidemiology, Mailman School of Public Health, Columbia University, New York, NY, United States

**Keywords:** opioid use disorder, stigma, buprenorphine, substance use services, medications for opioid use disorder

## Abstract

Stigma toward people taking medication for opioid use disorder (MOUD) is prevalent, harmful to the health and well-being of this population, and impedes MOUD treatment resource provision, help-seeking, and engagement in care. In recent years, clinicians have implemented new models of MOUD-based treatment in parts of the United States that integrate buprenorphine initiation into emergency departments and other acute general medical settings, with post-discharge linkage to office-based treatment. These service models increase access to MOUD and they have potential to mitigate stigma toward opioid use and MOUD. However, the empirical literature connecting these emerging service delivery models to stigma outcomes remains underdeveloped. This paper aims to bridge the stigma and health service literatures via a conceptual model delineating how elements of emerging MOUD service models can reduce stigma and increase behavior in pursuit of life goals. Specifically, we outline how new approaches to three key processes can counter structural, public, and self-stigma for this population: (1) community outreach with peer-to-peer influence, (2) clinical evaluation and induction of MOUD in acute care settings, and (3) transition to outpatient maintenance care and early recovery. Emerging service models that target these three processes can, in turn, foster patient empowerment and pursuit of life goals. There is great potential to increase the well-being of people who use opioids by reducing stigma against MOUD via these structural changes.

## 1. Introduction

### 1.1. Opioid use disorder and its treatment

Overdose remains a leading cause of death for Americans under 50 (1). In 2018, an estimated 10.3 million people (3.7% of the population over age 12) in the United States used opioids in a way not prescribed (2). The American Society of Addiction Medicine (ASAM) conceptualizes opioid use disorder (OUD) as a “treatable, chronic medical disease involving complex interactions among brain circuits, genetics, the environment, and an individual’s life circumstances” that is associated with increased risk of death, injury, and infections (e.g., HIV, hepatitis, endocarditis) (3). Clinical practice guidelines recommend combination pharmacotherapy (with methadone, buprenorphine, or extended-release naltrexone) and offering individualized psychosocial treatments for all individuals with OUD (3). Medications for opioid use disorder (MOUD) rapidly stabilize brain circuits affected by addiction, reducing craving and withdrawal symptoms, and enabling patients to engage in healthy changes that facilitate recovery (3). Methadone and buprenorphine maintenance treatments (MMT and BMT, respectively) significantly reduce illicit opioid use (4), mortality (5, 6), and risk for acquiring HIV (7). Non-MOUD approaches to treating OUD, such as detoxification or psychosocial treatments alone, are considerably less safe and effective (8). Despite strong evidence supporting MOUD and the widespread urgency of addressing the opioid epidemic, MOUD remain vastly underutilized. Worldwide, the World Health Organization has estimated that only 10% of individuals needing MOUD receive it (9). In the United States in 2014, only 41% of adults *entering treatment for OUD* received MOUD (10).

### 1.2. Stigma as a barrier to OUD treatment

Stigma is often implicated as a significant barrier to MOUD (11, 12). Stigma has been defined as the co-occurrence of labeling, stereotyping, separation, and status loss and discrimination in the context of a power differential (13), and has been proposed as a fundamental cause of health inequalities (14). Substance use and substance use disorders (SUDs) are highly stigmatized globally, particularly OUD (15). Stigma toward opioid use, OUD, and MOUD is perpetuated and reinforced by laws and policies (i.e., structural stigma), reflected in public attitudes toward people who use opioids (i.e., public stigma), and internalized by people who use opioids themselves (i.e., self stigma) (see Table 1 for definitions of key stigma concepts). Structural stigma is evident in a hostile policy environment that permits discrimination against people using opioids (e.g., loss of employment, housing, child custody) (17). Additional manifestations of structural stigma include the historical separation of OUD treatment from other healthcare, MMT treatment program regulations that created carceral rather than therapeutic treatment environments and reinforced the illegality of OUD, frequently-changing federal guidelines and insurance regulations that have discouraged providers from treating OUD, and a longstanding lack of training around OUD in medical education that has left most physicians and nurses unprepared and underconfident to effectively assist patients with OUD (11, 18–22). Structural stigma at the health facility level is also closely related to the stereotypes, prejudice, and discrimination held and enacted by clinicians (i.e., healthcare provider stigma) since health facility policies and procedures highly influence, and in turn can be influenced by clinicians and how they deliver health services. We differentiate between the policies within healthcare settings as forms of structural stigma, and the negative attitudes held by health care providers as an example of attitudes held by “stigmatizers” (below). As for public stigma toward opioid use, much of the research examines stigmatizing beliefs (e.g., that people with prescription OUD lack self-discipline and should be blamed for their condition) (23) and attitudes, such as low public support for harm-reduction methods (e.g., supervised injection sites and needle exchange programs) (24, 25) and dismissing people in recovery with MOUD as “replacing one addiction for another” (26, 27). Both structural and public stigma can then be internalized by people who use opioids, who have reported feelings of self-blame, self-loathing, despair, shame, and as if they have “permanently screwed up their lives” and are “out of place in the world.” (26, 28). Further, when the person who uses opioids carries additional identities that can serve as bases for marginalization (e.g., gender, race/ethnicity, and other medical or psychiatric conditions), self-stigma and its negative impacts can be worsened (26, 29, 30). Female, older aged, Black, Latinx, pregnant, and low-income individuals are some sociodemographic groups made to face even steeper barriers to treatment (31–33).

**Table 1:** Definitions of key stigma concepts.

Locus	Concept (alternative name for concept)	Definitions	Impact on health services
Society	Public stigma (Societal Stigma)	Widely shared negative beliefs about members of a particular group	Low public support for harm reduction services (e.g., “not in my backyard” attitudes toward clinics that provide substance use services, low support for harm reduction services, such as safe injection sites)
	Structural Stigma (includes *health facility stigma*)	Societal conditions, such as laws, policies and institutions (e.g., healthcare), that limit choices, resources, and well-being for members of a group	Unequal access to treatment; Limited attainment of psychosocial outcomes (e.g., employment)
Stigmatizers (e.g., healthcare providers, family members)	Stereotypes	Cognitive response- beliefs about characteristics and behaviors of certain group of people (e.g., dangerous, untrustworthy)	Lead to discriminatory behavior by healthcare workers
	Prejudice	Affective response- feelings toward a member of a group (e.g., fear, pity, anger)	Can lead to nonverbal signs of devaluing the patient by healthcare workers
	Discrimination	Behavioral response- unequal behavior directed at a member of a group (e.g., avoidance, withholding, coercion)	Harsh treatment, negative patient experiences in health care settings
Stigmatized	Anticipated Stigma (Felt stigma)	Degree to which a stigmatized individual expects to be the target of stereotypes, prejudice, or discrimination in the future (e.g., worrying about being treated unfairly)	Avoidance of treatment
	Self-stigma (internalized stigma)	Extent to which one applies stigmatizing beliefs and feelings to themselves (e.g., blaming oneself for one’s illness, feeling incompetent)	Low self-esteem, psychological distress, symptom severity, maladaptive coping behaviors (e.g., including perpetuating substance use)

Definitions derived from Hatzenbuehler et al. (14) and Fox et al. (16).

### 1.3. The emergence of new MOUD models

From 2004 to 2015, emergency room visits in the United States related to OUD doubled and medical hospitalizations increased 64% (34), motivating new initiatives aiming to proactively treat OUD in acute medical settings, which have shown initial promise in closing the MOUD treatment gap (35). Over the past decade, clinicians, researchers, and advocates have described new models for starting and continuing MOUD in emergency departments and inpatient settings (36). These emerging models have a bidirectional relationship with stigma (11, 12). First, independent efforts outside of these models to reduce structural, public, institutional, provider, and patient (self) stigma can improve implementation of these emerging models and can increase uptake of MOUD. This has the potential to both improve patient outcomes and to reduce acute health service utilization. Second, emerging models for MOUD may themselves directly reduce stigma by acting on distinct stigma processes that can occur at each stage of OUD treatment, as we will describe in this paper. By leveraging their anti-stigma potential, these emerging treatment models can address OUD-related stigma at the institutional, provider, and patient levels.

### 1.4. Using the “Why Try” model to identify how emerging MOUD models interact with stigma

**Figure 1:**
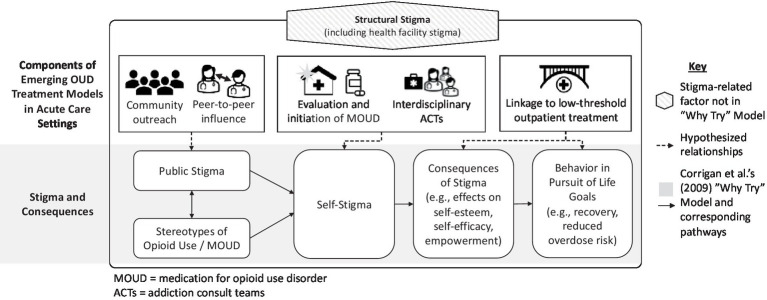
Depiction of emerging models of OUD treatment as they relate to the “Why Try” model.

To identify the potential stigma implications at each stage of OUD assessment and treatment initiation, we draw on Corrigan’s (2009) model of the “Why Try” effect (37). The “Why Try” model proposes that individuals follow a three-step process in developing self-stigma: (i) awareness of stereotypes, (ii) agreement with stereotypes, and (iii) application of stereotypes to oneself (i.e., self-stigma). This process, in turn, leads to low self-esteem and self-efficacy, which subsequently interfere with pursuit of treatment and other life goals. For example, an individual might think, “Why bother trying to pursue any life goals and treatment if I view myself as a worthless and undeserving addict” because of the stereotypes absorbed and applied to oneself (see Figure 1).

Here, we propose a conceptual framework describing how particular forms of stigma are associated with key phases of MOUD service delivery. This framework provides a structure for evaluating the relationships between emerging service models and stigma and for developing new stigma-reduction strategies. Specifically, we describe how emerging models could counteract self-stigma and the “why try” effect through the following three processes: (1) community outreach and peer-to-peer influence, which can reduce public stigma; (2) inpatient evaluation and initiation of MOUD, which can reduce structural (e.g., health facility) stigma by changing clinical procedures and shifting healthcare worker attitudes and self-stigma by starting treatment; and (3) transitions to post-discharge care, which can mitigate the negative impact of stigma broadly by increasing engagement in recovery-oriented behaviors.

We first provide an overview of emerging models of MOUD initiation in acute care settings developed in the United States. Next, we describe how the three processes described above (community outreach, inpatient evaluation and initiation of MOUD, and transitions to post-discharge care) intersect with public stigma, self-stigma, and the consequences of stigma. We focus here on acute care initiation of MOUD, rather than outpatient programs, because emergency departments and hospital wards have been major sites of SUD service innovation, to treat the high proportions of patients presenting to hospitals with co-occurring SUDs over the past decade of an unrelenting opioid crisis in the US (36). For each process, we describe the current situation (the interplay of stigma and longstanding models of OUD treatment), we discuss the potential ways emerging models could mitigate stigma, and we propose hypotheses to inform how future research could evaluate the impact of emerging models on stigma. Since relatively little empirical work has examined how MOUD initiation changes public stigma or the consequences of stigma, these sections are more speculative at this time.

## 2. Emerging models of proactive OUD treatment

In this section we will describe the structure, processes, and some preliminary evidence for emerging OUD acute service models, to provide background for our analysis (in Section 3) of how implementation of these models may reduce OUD-related stigma.

### 2.1. Historic OUD treatment models

Structural stigma has pervaded management of patients with OUD in hospital settings, with patients with OUD receiving treatment and care coordination for their SUD well below the standards of other medical conditions. Historically, the standard of care for addressing SUDs in general medical settings (e.g., emergency departments, medical wards, primary care offices) has been met simply by providing patients identified as having substance use problems a list of substance use clinics, ultimately requiring patients to self-present elsewhere for substance use treatment (38). MOUD remain underutilized, likely in part because these traditional pathways to MOUD delay treatment initiation and present patients with several obstacles such as sufficient knowledge about treatment options, concerns about ability to afford treatment, and the anticipated stigma of connecting oneself to a treatment site (38). Efforts to promote structural changes that reduce these longstanding and widespread stigmatizing practices are urgently needed and beginning to emerge.

### 2.2. Emerging MOUD models

A host of novel methods have emerged over the past decade to raise the standard of care for people with OUD in acute medical settings (see Table 2 for overview) (36). These include interprofessional addiction consult services that provide evaluation and treatment for SUDs, support medical needs, and promote systems change; psychiatry consultation-liaison services that may offer motivational interviewing and/or MOUD as well as support management of co-occurring psychiatric disorders; individual consultants that help provide MOUD and linkage to aftercare, and integration of MOUD into primary team services (e.g., hospitalists or emergency medicine physicians routinely prescribing buprenorphine) (36).

**Table 2:** Examples of emerging acute care models of substance use treatment.

Model	Team composition	Main activities
Addiction consult team models: specialists assist in evaluation and treatment planning	Interprofessional addiction consult teams (ACTs): often include an addiction specialist (physician or advanced practice provider), social worker or case manager, may include peers, nurses, or pharmacists	- Comprehensive evaluation of patients who use substances - Engagement efforts (e.g., peers), harm reduction approaches (e.g., fentanyl test strip education) - Treatment: withdrawal management, medications for addiction treatment, support and advocacy for acute medical needs (e.g., heart valve surgery) - Discharge planning including pathways to community treatment and addressing social determinants of health - System-focused: staff education, addressing stigma, influence hospital policy and practices
	Psychiatry consult-liaison services: usually includes a psychiatrist (may or may not have addiction fellowship training) and social worker or psychologist with expertise in local mental health resources	- Comprehensive assessment of co-occurring psychiatric and substance use problems - Treatment: May offer motivational interviewing or cognitive behavioral therapy, management of psychiatric medications, may or may not offer medications for opioid use disorder (MOUD) - Assist with recommendations about post-hospital treatment (e.g., inpatient vs. outpatient programs)
	Individual consultants: addiction specialist physician (may have background in internal or family medicine, psychiatry, toxicology, etc.)	- Assess substance use - Treatment: assist with withdrawal management, offer medications for addiction treatment, provide “bridge” prescription at discharge - Partner with primary team (e.g., social worker) for referrals - System-focused: may help develop protocols and education materials
Practice-based models: primary team clinicians (e.g., hospitalists or emergency physicians) offer medications for addiction treatment as part of their usual practice	General hospital providers (e.g., hospitalists, infectious disease specialists, residents) with buprenorphine training, often provided with ongoing clinical supports (e.g., mentoring, warm lines, trainings)	- Offer MOUD based on protocols and order sets, sometimes provide naloxone kits and overdose education - Provide “bridge” prescription of MOUD at discharge and referral to community-based treatment
In-reach models: community-based clinicians provide remote treatment to hospitalized patients with linkage to community care after discharge	Community-based providers who manage MOUD- usually primary care physicians or addiction specialists	- Provide brief assessment via video or phone - Offer guidance to primary teams about management of MOUD - May offer “bridge” prescription and follow-up appointment after discharge

Descriptions adapted from Englander et al. (36).

One emerging model to raise the standard of care for people with OUD and mitigate structural stigma is to initiate MOUD in emergency department (ED) settings. People using opioids frequently present to hospitals for related issues, such as injuries, overdose, or infections (e.g., endocarditis, soft tissue infections) (39). For many patients, EDs may be their only contact point with medical providers, creating opportunities for initiating MOUD with fewer delays and barriers than traditional pathways to care. A landmark RCT found that ED-based buprenorphine initiation (with linkage to primary care follow-up) for OUD significantly outperformed traditional interventions [e.g., referral or screening, brief counseling intervention with referral (SBIRT)] in increasing treatment engagement at 30-days and 2 months, reducing self-reported illicit opioid use, and decreasing utilization of inpatient detoxification services (40, 41). Subsequent analysis additionally found ED-based buprenorphine induction cost-saving for health care systems and time-saving for patients, compared to traditional approaches (42). ED-based buprenorphine initiation has yet to be widely adopted. Structural barriers such as inadequate physician preparation and availability of referrals for ongoing treatment after ED discharge (due to the lack of integration of SUD treatment into traditional healthcare systems) have limited implementation (43, 44).

Other models in general hospital settings such as addiction consult teams (ACTs) have also shown promise in improving MOUD initiation and reducing OUD-related stigma among hospital staff. Multidisciplinary addiction consult teams (e.g., addiction physician, social worker, peer recovery coaches) assist with evaluation and medication initiation, provide education and brief interventions, including harm-reduction, and coordinate linkages to aftercare (36, 45–47). Treatment of SUDs underlying medical problems have generally been sub-optimally addressed in general medical settings, with MOUD seldom part of the discharge plan (48). Detoxification alone leaves patients vulnerable to overdose after discharge. An RCT enrolling hospitalized patients with OUD who were not seeking MOUD, found that initiation of buprenorphine maintenance therapy (BMT) with linkage to primary care significantly outperformed detoxification in engaging patients outpatient BMT and reducing illicit opioid use at 6 months (49). ACTs in some centers have also begun expanding MMT initiation in hospitals, a practice that has historically been hampered by misunderstandings about the legality of starting MMT in hospitals (50, 51).

Addiction consultants may also assist in management of withdrawal symptoms and pain and can facilitate hospital SUD policy development and promote cultural and structural change (47). For individuals with OUD who are admitted to medical or surgical wards for treatment of acute problems, withdrawal symptoms can create distress and interfere with management of acute medical problems (49). Traditionally, opioid withdrawal has been treated with a methadone or buprenorphine detoxification taper or non-opioid medications (e.g., clonidine) (52). Addiction consult teams are also guiding implementation of new approaches to safely manage withdrawal, such as initially using escalating doses of scheduled short-acting opioids to treat from fentanyl before transitioning to methadone or buprenorphine (53). When successfully established (i.e., teams are equipped to assist with assessment, withdrawal management, MOUD initiation, counseling, peer engagement, and linked to community-based care), addiction consult teams can also reduce stigma among hospital staff, improving attitudes toward patients with SUD and improving perceptions the SUD are treatable (54). Hospitalists with access to support from addiction specialists are more likely to screen patients for OUD, initiate treatment, and endorse feeling supported by their institution in caring for patients with OUD (55) and involvement of addiction consultants has been associated with increased 30-day abstinence and decreased addiction severity (46). However, barriers to these models still exist; SUD- and OUD-related stigma among clinicians and hospital leadership has limited successful implementation of addiction consult teams (56).

## 3. How structural changes in OUD management interact with stigma processes across the MOUD treatment cascade

In the following sections, we draw from the “Why Try” framework to examine stigma at each phase of MOUD treatment delivery (Figure 1). We start with (1) pre-hospital settings (e.g., community outreach and emergency medical services), before moving onto (2) hospital-based evaluation and treatment, and ending with (3) transition to long-term outpatient services. We draw from existing literature describing stigma processes in both traditional healthcare system management of OUD and in emerging models, to compare how changes might mitigate stigma at each stage of MOUD delivery. See Table 3 for an overview of each stage, focusing on how structural factors shape self-stigma and patient behavior.

**Table 3:** Interplay between emerging treatment models and related stigma processes.

Phases of “Why Try”	Traditional pathways to care: structural stigma and related stigma processes	Emerging models: structural changes and potential impact on related stigma processes
(1) Community outreach and peer influence *(associated with public stigma)*	- **Structural stigma:** current outreach very much focused on raising awareness of OUD as a problem, dangerousness of opioid use; over-separation of addiction treatment into specialized clinics apart from general health facilities - **Public stigma:** OUD is dangerous, immoral, hard to treat, treatment (i.e., MOUD) is stereotyped as another form of social unacceptable addiction	- **Structural changes:** health department and health facility partnerships, shift messaging from dangerousness to the existence and effectiveness of MOUD and where to get it - **Changes in public stigma:** increased knowledge of efficacy of MOUD, capacity for recovery from OUD, and where to get MOUD
(2) Evaluation and initiation of treatment in general medical settings *(associated with self-stigma)*	- **Structural and healthcare provider stigma**- healthcare provider (HCP) stereotypes of people with OUD: violent, manipulative, poorly motivated, unreliable, incompetent. HCP enacted stigma: lack of respect for patient autonomy, non-collaborative treatment planning, punitive care terminations, dosage or duration caps, treatment interruption - **Anticipated and self-stigma:** people with OUD are aware of stigma among HCPs, feel marginalized, resulting in avoiding medical settings, avoiding disclosing opioid use to HCPs, delaying MOUD treatment	- **Structural changes:** Initiatives that empower and support physicians in treating OUD can change conceptualizations of OUD as a disease, improve physician attitudes, and improve quality of care for people with OUD, in a harm reduction framework; patients provided multiple options as an active participant in shared treatment decision making - **Changes in anticipated and self-stigma and treatment engagement:** experiences in EDs are improving with new initiatives, new approaches improve people with OUD’s trust in medical providers, patients respected as active participants in treatment decisions with shared responsibility
(3) Transition to long-term outpatient treatment and early recovery *(associated with self-efficacy and behavior in pursuit of life goals)*	- **Structural Stigma:** patients provided a list of outpatient clinics with minimal treatment or education about evidence-informed options and left to follow-up on their own - **Self-efficacy/behavior in pursuit of life goals:** perceive that they should handle OUD alone, denial of OUD as a medical problem, pursue treatments with weaker evidence base that may be harmful (e.g., inpatient detoxification)	- **Structural changes:** linkage from acute care to outpatient treatment, flexible low-threshold clinic structures, supportive and non-judgmental staff stance, individualized and patient-centered, involvement of peer recovery coaches to reduce power differentials and enhance motivation for change - **Changes in self-efficacy/behavior in pursuit of life goals:** empowered, improved treatment adherence, increase in other behaviors in pursuit of life goals (e.g., work, relationships)

### 3.1. Community outreach and peer-to-peer influence: associated with public stigma

#### 3.1.1. Current situation

Traditional pathways to treatment (e.g., opioid treatment programs that dispense methadone) can reinforce public stigma because of their separation from mainstream healthcare services and lack of integration with community anti-stigma campaigns. Although the separation of specialized addiction treatment gives patients access to a concentration of expertise, it also allows for social observation and labeling of individuals who attend these clinics, making them more susceptible to negative public attitudes (18, 27, 32). This relationship is likely bidirectional: public stigma and its internalization (i.e., self-stigma) are linked to lower rates of specialized OUD care engagement, while at the same time lower rates of specialized OUD care increases the chance that people with OUD will continue using opioids, thus confirming negative stereotypes (29, 57). Additionally, providers in specialized clinics are likely to interact primarily with peers who also operate within these clinics, limiting the potential for peer-to-peer attitude change. Moreover, typical pathways to MOUD treatment are rarely integrated into OUD-related community-based campaigns or outreach activities, such as those that aim to reduce stigma of OUD, represent lived experience of people with OUD, and promote hopeful messages about treatment generally [e.g., New York City’s *Living Proof* campaign (58) and Colorado’s *Lift the Label* (59)]. This could in part be due to the public’s prevalent negative attitudes toward MMT and other current common models of addiction treatment such as those reflected in decades of negative news stories that focus on the problems associated with MOUD, the relative prevalence of negative depictions of people who use opioids or are taking MOUD (e.g., as being more prone to violence), and longstanding beliefs about the moral failing of people with OUD (23, 60, 61).

#### 3.1.2. New approaches

Aspects of emerging acute care service models for MOUD treatment—such as their setting and staffing—can circumvent some of the public stigma that prevents patients from seeking OUD care in specialized settings. In at least one case, emergency medical services personnel have been trained in providing harm-reduction-focused education, motivational interviewing, and MOUD treatment to people with OUD in the pre-hospital setting (62), which may help engage people who avoid presenting to the hospital altogether in efforts to avert stigma and discrimination. When patients with OUD present to acute care settings with any medical concerns, trained clinicians in new treatment models have the opportunity to screen, identify, and evaluate patients’ OUD treatment needs. The fact that this occurs in a generalized, acute care setting rather than a specialized clinic means individuals will be able to being engagement with OUD treatment without facing the barrier of being seen going to addiction treatment and assigned the corresponding negative stereotypes.

However, these alternative pathways to care are unlikely to serve as a panacea to all public stigma, and there is great promise in increasing integration between these new models of MOUD treatment and public stigma campaigns. Although these models offer an opportunity for people with OUD to avoid the full brunt of stigmatization and may readily result in stigma change within acute care settings, they currently do little to change the persistent negative *public* attitudes around OUD. The studies identified by a 2020 review of “non-traditional” buprenorphine treatment models as using “community outreach” [e.g., via a mobile syringe exchange program (63), posting flyers in community spaces (64)] used community outreach solely as a recruitment strategy (38). Ideally, such community outreach would be implemented alongside widespread, effective public education campaigns that incorporate lived experience narratives and center on OUD being a treatable medical condition, how to access MOUD, and MOUD’s effectiveness (65). These sorts of campaigns are urgently needed, as stigmatizing language use in news media is increasing (61), and existing campaigns often overfocus on statistics that communicate the scope of the crisis, not its solutions (66).

#### 3.1.3. Hypotheses

We propose the following hypotheses, that could be tested while implementing anti-stigma efforts in conjunction with new service models or community-based anti-stigma campaigns:

- Community outreach for MOUD may be improved by partnership with local public health departments to concurrently roll-out anti-stigma campaigns.
- Campaigns that emphasize that recovery from OUD is possible, buprenorphine and methadone are effective treatments for OUD, and where and how to access buprenorphine and methadone in the community may decrease stigma and increase MOUD utilization.

### 3.2. Inpatient evaluation of OUD and management of OUD: associated with self-stigma

#### 3.2.1. Current situation

Stigma within healthcare settings negatively impacts patients with OUD. Stigmatizing beliefs among healthcare staff translate into negative attitudes and unequal treatment. A systematic review described commonly-held stereotypes among healthcare providers (HCPs) of people with SUDs as violent, manipulative, and poorly motivated, associated with generally negative attitudes toward these patients (67). When HCP stigma becomes enacted, it can result in lack of respect for patient autonomy, non-collaborative treatment planning (e.g., around taper schedules), punitive care terminations (e.g., discharge for policy violations or positive urine toxicology results), imposition of dosage or duration caps, which can lead to treatment interruption or avoidance, ultimately exacerbating the treatment gap (68).

These explicit manifestations of structurally embedded stigma can directly become internalized as self-stigma. In a Canadian study of individuals on MOUD, HCP were identified as the second most common source of stigma after friends (69). Individuals with OUD have described facing elevated scrutiny from HCP who were concerned they were exaggerating symptoms to obtain opioids and expressed perceptions that they were viewed as “junkies,” trying to get high, incompetent, unreliable, and lacked willpower (69, 70). Half of the Canadian respondents agreed with some of these stereotypes (i.e., internalized them as self-stigma), and half also endorsed feeling ashamed of taking MOUD, leading them to question their decision and feel depressed (69). Meanwhile, efforts to navigate self-stigma can exacerbate negative health-related behaviors, undermine efficacy of interventions, and lead to worse health outcomes (71). In efforts to avoid stigma, respondents endorsed delaying MOUD treatment, lowering their dose prematurely (leading to withdrawal symptoms), and avoiding EDs and primary care visits unless in extreme pain (69). To avert stigma, individuals taking MOUD or using opioids sometimes choose not to disclose their use to healthcare providers, which could lead to adverse outcomes, such as physicians prescribing them medications that can cause fatal interactions and reduced opportunities for appropriate support (32). Poor overall health status in opioid users has been attributed in part to stigma in healthcare settings (72).

#### 3.2.2. New approaches

Early evidence suggests the emergence of ED-based OUD treatment may be reducing HCP stigma in EDs and improving patient engagement, which may reflect reductions in self-stigma. Focus groups of patients with OUD across four US cities described historically feeling stigmatized by treatment in ED settings, with HCP not perceiving OUD as a medical disease, leading to dehumanizing experiences with pain and other unmet medical needs due to their OUD histories (73). However, these patients also noted recent improvements in emergency care of patients with OUD, and positive experiences with some providers (73). Emerging models of acute SUD treatment (e.g., ED-initiated buprenorphine initiatives, addiction consult teams) that have emphasized proactive treatment of OUD in acute settings may reduce HCP stigma by empowering physicians to care for patients with OUD—evidence has suggested that emphasizing the treatability of conditions can mitigate stigma (74). Ongoing training and support appear critical to empowering physicians to improve treatment of OUD and reducing HCP stigma. A qualitative study of ED physicians identified three facilitators to physicians starting buprenorphine in the ED, including (1) knowledge about OUD and buprenorphine, (2) positive experiences prescribing buprenorphine in the past, and (3) local physician champions to promote the practice (75). These studies emphasize the importance of ongoing ED-based continuing medical education on OUD as a disease, buprenorphine treatment protocols and clinical decision support tools (e.g., sets of physician orders including vital signs, withdrawal symptom monitoring, labs, and medications necessary for management of opioid withdrawal), and involvement of allied professions to help with counseling and post-discharge follow-up (73, 75).

Similarly, early evidence suggests that addiction consult teams (ACTs) can reduce HCP stigma by providing physicians with education and specific recommendations to help them provide more effective treatment for OUD and improve patient experiences on inpatient wards, which may reduce self-stigma among patients. As observed in a qualitative study, the presence of addiction consultants validates OUD as a medical problem aligned with conditions managed by other specialized consultation services (47). A survey of hospitalists found that support from ACTs positively impacts quality of addiction-related treatment by hospitalists (55). ACTs assist with assessment of SUDs, provide education to patients and primary clinicians, assist with withdrawal management and MOUD initiation, and facilitate appropriate arrangement of aftercare (45). These services can facilitate cultural change by improving HCP attitudes about OUD, reducing negative stereotypes, and shifting clinician behavior from discriminatory practices toward patient empowerment (54). Some ACTs include peers in recovery as part of the team—contact with narratives of people in recovery from OUD can enhance messages about treatment effectiveness and further reduce stigma among clinicians (76). Contact with peers, whose stories model recovery, can provide hope for both patients with OUD and their clinicians, and has been found as one of the most effective anti-stigma approaches among healthcare professionals, eliciting more positive attitudes and acceptance, but nonetheless remains widely underused (66).

Providers in one hospital, before implementation of an ACT described medical hospitalizations not addressing addiction, with beliefs that withdrawal could not be treated, misperceptions about legality of starting MOUD in the hospital, or perceived impropriety of treating addiction instead of pain (54). Lack of treatment led to patient discomfort from withdrawal, disruptive behavior, and against medical advice (AMA) discharges, ultimately leading to HCP feelings of futility, moral distress, and burnout. Following the implementation of an ACT, HCP attitudes and treatment of patients with SUD changed dramatically. ACT members effectively educated HCP about addiction as a disease, modeled communication and compassionate care, and empowered physicians to properly diagnose and treat patients with OUD (54).

Increased attention and resources devoted to counseling patients about OUD and educating them about treatment options for underlying SUDs of acute medical issues can emphasize the conceptualization of SUD as a legitimate medical issue and show respect for the patient’s needs. A patient-centered approach to initiation of MOUD in acute settings, providing patients with information and treatment options to tailor treatment to their personal needs, in the context of trust, empathy, and mutual respect, can set the stage for further engagement with outpatient MOUD services. Patients with OUD have expressed a strong preference for SUD services that cultivate a patient-focused orientation, respect in clinical settings, shared power and responsibility for treatment decisions, and a supportive environment for recovery (77). Among hospitalized patients seen by an ACT, trust in hospital-based doctors increased, which was attributed to ACTs utilizing a nonjudgmental and compassionate approach, fostering agency, establishing their reliability (i.e., showing themselves to be dependable and true to their word, such as following through after discussions about treatment plans), and providing treatment that was effective in meeting the patient’s acute needs (e.g., withdrawal) (78). By reversing HCP stigma, the initiatives above would be expected to reverse self-stigma among patients with OUD that has been reinforced by historically stigmatizing treatment in healthcare settings, but this remains to be comprehensively systematically examined.

#### 3.2.3. Hypotheses

We propose the following hypotheses, that could be tested while implementing new service models to assess impacts of structural changes on stigma and its impacts:

- Improved HCP knowledge about OUD- conceptualizing OUD as a *treatable* disease (vs. a moral weakness or willful choice), will reduce negative stereotypes and discrimination related to people with OUD.
- Improved physician knowledge of the efficacy of MOUD and comfort prescribing MOUD will reduce discrimination (e.g., non-collaborative treatment planning, insufficient dosing, punitive treatment).
- Clinician contact with peer specialists embedded in ACTs will reduce HCP stigma toward people with SUDs.
- HCP will perceive less stigma in medical settings where MOUD initiation is normalized and physicians are adequately trained and supported (e.g., consultation available from ACTs).
- Lower HCP stigma will result in lower self-stigma among patients for with OUD.
- Lower self-stigma will lead to more patient willingness to access health services, disclose their substance use history to HCP, which will minimize adverse events related to medication interactions, improve adherence, and improve health outcomes.

### 3.3. Transition to post-discharge and early recovery: associated with recovery-oriented behaviors that may mitigate stigma

#### 3.3.1. Current situation

Following identification, evaluation, and initiation of treatment for OUD, the next phase of recovery involves ongoing participation in treatment. Substance use treatment aims to aid patients in controlling symptoms so they can engage in productive, non-substance use-related activities (3). In the final phase of Corrigan’s “Why Try?” model, participation in evidence-based practices corresponds with recovery-oriented behaviors (e.g., re-entering the workforce, strengthening relationships) (37). Corrigan conceptualizes empowerment as an obverse to self-stigma, associated with active engagement in treatment and high self-esteem, quality of life, and social support (37). If a consumer feels disempowered, he’s less likely to attend clinic services or work toward other life goals. Enhancing user empowerment, through collaborative and self-directed services, should improve treatment engagement and attainment of recovery-oriented goals.

Currently, service models end with low engagement in evidence-based practices, such as MOUD maintenance treatment. Historically, patients who present to acute medical settings with OUD are provided a list of clinics and asked to follow-up on their own. Referrals lists may include medical detoxification facilities or abstinence-based rehab centers, which are not evidence-based treatment options. If patients are not counseled about evidence-informed recommendations for MOUD-based treatments, they may pursue no treatment or treatment that may ultimately be harmful. In an RCT, only 12% of patients who received traditional detox and referral treatment on a medical floor engaged in post-discharge treatment (49).

In one review, stigma was identified as one of the top three barriers to treatment seeking among substance users, with “should handle alone” and denial of a problem also frequently mentioned across studies (29). All three of these barriers may be perpetuated by the traditional management of OUD in acute medical settings—the lack of active management may exacerbate denial while the passive referral process may feed the perception that the user should manage the issue on their own. To our knowledge the relationship between stigma and retention in MOUD treatment has not yet been directly examined (68). However, consumers who do engage long-term in treatment can succeed in achieving life goals. A study of 12-year follow-up of patients taking MOUD, consistent treatment use was strongly associated with long-term recovery, which was associated with continued education, employment, more housing stability, and fewer marital transitions (79), aligning with Corrigan’s theory that participation in evidence-based practices is associated with achievement of life goals (37).

#### 3.3.2. New approaches

Emerging service models may better empower patients to engage in evidence-based practices and recovery. In RCTs, when buprenorphine was initiated in acute settings with linkage to outpatient follow-up, 78% of ED patients remained in treatment at 30 days (vs. 37% with traditional approaches) (40) and 72% of admitted patients engaged in outpatient follow-up (vs. 12%) (49). Low threshold clinic models have been developed to address concerns that traditional treatment programs are experienced as unwelcoming and stigmatizing by some patients (80). Low threshold models emphasize engagement and aim to be accessible to patients who have difficulty meeting expectations of traditional clinics (e.g., expectations for abstinence, rigid adherence to appointment times). In a qualitative evaluation of a hospital-affiliated low-threshold transitional clinic integrated with new approaches to acute initiation of MOUD, users identified the clinic staffs’ supportive and nonjudgmental attitudes, flexible clinic structure, and harm-reduction emphasis, as facilitators of continuing treatment, compared to traditional less patient-centered service models (80). Shared decision-making, with enhanced consideration of patients’ preferences and values, has been associated with increased patient empowerment in mental health settings (81). Patient empowerment and self-efficacy can be further augmented by integrating peer recovery coaches into outpatient treatment (82). Recovery coaches can empower patients by reducing the power differential between patients and clinic staff, sharing experiences, and enhancing motivation for behavioral change, and helping patients address social determinants of health (83).

#### 3.3.3. Hypotheses

We propose the following hypotheses, that could be tested while implementing new service models to assess impacts of structural changes on the impacts of stigma and recovery outcomes:

- Self-stigma is associated with low MOUD maintenance treatment adherence.
- Reduced self-stigma is associated with increased patient empowerment.
- Patient-centered care models (e.g., flexible, supportive, respectful, shared decision-making) will be associated with greater empowerment and greater treatment adherence.
- Integration of peer recovery specialists in outpatient treatment is associated with patient empowerment.
- Patient empowerment is associated with maintaining treatment adherence.
- Adherence to MOUD will be associated with greater attainment of life goals.

## 4. Discussion and conclusion

Emerging models of MOUD initiation in acute care settings have begun to transform treatment of OUD in some parts of the US. These models, rooted in a harm-reduction approach, emphasize low-threshold, flexible initiation of MOUD to improve access to effective treatment for OUD among patients presenting to hospitals for health concerns that may or may not be directly connected to their OUD. These structural transformations reflect a growing effort in SUD treatment to move away from a “tough on drugs” paradigm that can consider stigma as a tool to motivate behavioral change toward a harm reduction paradigm (84). In a harm reduction paradigm, stigma is viewed as morally unacceptable and also a driver of negative effects of substance use, especially among the most marginalized patients (84). OUD treatment in the US has historically been plagued by stigma in the pre-hospital setting (i.e., public stigma), healthcare settings (i.e., structural stigma), and among individuals who could benefit from treatment (i.e., self-stigma). Self-stigma can lead to worsening mental health and substance use, impede accessing treatment, and limit attainment of life goals (85). As this paper has sought to highlight, these three levels of stigma are also inextricably linked and intervening on any one level will thus necessarily impact the others, and intervening at all levels simultaneously could be most effective (86). Prior research has demonstrated how structural change (e.g., provision of effective evidence-based treatment) can reduce public and self-stigma related to other health conditions (87, 88). Although efforts to target stigma at the individual level directly (e.g., self-stigma and stigma held by clinicians) may be helpful, allotting time and resources toward addressing public and structural stigma are critical elements of improving engagement in effective treatment and reducing rates of opioid overdose in the US. The structural transformations involved in hospital-based management of OUD are a promising approach to addressing stigma across all three of these levels by interfacing with campaigns to mitigate public stigma, mitigating the impacts of historic and ongoing structural stigma (particularly in healthcare settings), and creating a health-promoting, judgment-free environment for those who choose to seek treatment that does not generate such high levels of self-stigma as traditional approaches.

Numerous studies have evaluated the effectiveness of new models of treatment (e.g., ED-initiated or hospital-initiated buprenorphine) on engagement in MOUD treatment, with promising short-term results (40, 41, 49). Although these innovations represent a substantial advance from prior treatment models, absolute levels of medium-to-long term engagement remain low (e.g., no significant differences compared to traditional referral at 6 and 12-months) (41) and long-term psychosocial recovery outcomes remain under-investigated. Stigma likely plays a role in engagement, although direct relationships between stigma and MOUD treatment engagement have not been thoroughly investigated (68). In order to maximize the effectiveness of treatments and improve outcomes that matter to patients [e.g., “live a normal life,” control cravings/withdrawal, “get clean,” and maintain stable employment (89, 90)] further empirical investigation is needed to better understand the role of stigma throughout treatment cascades (91).

To better understand the effect of new innovations (i.e., structural changes) on stigma among providers and patients, further research is needed to assess the provider and patient experience with new models of care. For example, although qualitative studies suggest that new models may be reducing stigma among healthcare providers and patients (54, 78), further studies can assess the impact of exposure to new treatment models on self-stigma (e.g., patient beliefs about themselves due to having OUD) and patient behavior (e.g., avoidance of treatment settings due to stigma), to better delineate the role of stigma processes on patient engagement/outcomes throughout treatment cascades. Although OUD-specific stigma scales are still relatively limited compared to those for other conditions (e.g., mental illnesses, HIV), they have rapidly proliferated in the past several years, particularly for assessing stigma at the individual and public levels (92). Use of these existing scales along with continued development and validation of opioid-related stigma scales could help to identify where stigma remains most severe, as a means to target subgroups with evidence-based stigma interventions. For example, if providers were surveyed, and high levels of stigma were identified on specific services, these services could be engaged in contact-based interventions (93) and educational messaging (76) to reduce stigma and provided additional support from ACTs to facilitate effective treatment of OUD, which itself may be a transformative contact-based experience for clinicians (94). If subgroups of patients with high levels of OUD stigma were identified (e.g., patients facing intersectional stigma on the basis of multiple marginalized identities), this would enable further exploration of structural factors and individual-level correlates that could be leveraged to better empower these patients and engage them in care. Moreover, little research to date has sought to develop means of quantifying opioid-related stigma at the institutional and structural levels. Quantification of structural stigma related to opioids is also important to identify, intervene on, and evaluate the effects of interventions targeting these pervasive macro-level forces.

The framework and hypotheses presented in this paper should be considered in the context of several limitations. This article has focused on literature drawn from North American healthcare settings and thus may or may not apply to healthcare systems in other parts of the world. We have focused on acute care services, to analyze the potential impacts on stigma of major innovations in substance use services that have developed over the past decade. However, future work must also consider how the structure and processes of various forms of outpatient services might impact patient experiences and stigma, since much of substance use treatment occurs in outpatient settings. Finally, although we have hypothesized mechanisms by which emerging models of OUD treatment in acute settings may reduce stigma, it remains possible that these changes could have unintended consequences and instead *increase* stigma in some circumstances. For example, more proactive efforts to screen for OUD would be expected to increase identification of patients with OUD, but having more patients labeled with OUD (e.g., in the medical record) might subject more patients to discrimination by some healthcare workers. Additionally, system-wide efforts to increase identification and treatment of OUD may subject clinicians to compassion fatigue, and subject patients to negative reactions (e.g., resentment) among clinicians who do not view management of OUD as part of their role. These risks would likely be most severe in settings in which limited resources, training, and support are provided to clinicians who are asked to take on new roles in addressing OUD.

Stigma is a widely identified barrier to MOUD treatment initiation and continuation, but its role in patient attrition throughout treatment cascades is not yet well studied. We provide a theoretical framework, extending from Corrigan’s “Why Try” model of mental illness stigma, to hypothesize how specific stigma processes interact with each stage of MOUD treatment. Further research examining these hypotheses can improve our understanding of the role stigma plays in treatment outcomes, and identify areas to target in order to reduce stigma, improve patient engagement, and facilitate patients’ attainment of psychosocial outcomes that “matter most” to them (95).

## Data availability statement

The original contributions presented in the study are included in the article/supplementary material, further inquiries can be directed to the corresponding author.

## Author contributions

TB contributed to conceptualization, investigation, visualization, writing—original draft, writing—review and editing. EE contributed to conceptualization, investigation, visualization, writing—original draft, writing—review and editing. AT contributed to writing—review and editing. LY contributed to conceptualization, supervision, and writing—review and editing. All authors contributed to the article and approved the submitted version.
